# Heavy Resistance Training in Hypoxia Enhances 1RM Squat Performance

**DOI:** 10.3389/fphys.2016.00502

**Published:** 2016-11-03

**Authors:** Mathew W. H. Inness, François Billaut, Emily J. Walker, Aaron C. Petersen, Alice J. Sweeting, Robert J. Aughey

**Affiliations:** ^1^Institute of Sport, Exercise and Active Living, Victoria UniversityMelbourne, VIC, Australia; ^2^Western Bulldogs Football ClubMelbourne, VIC, Australia; ^3^Département de Kinesiologie, Université LavalQuebec City, QC, Canada; ^4^Collingwood Football ClubMelbourne, VIC, Australia

**Keywords:** hypoxia, resistance training, strength, 1RM squat, power, hypertrophy

## Abstract

**Purpose:** To determine if heavy resistance training in hypoxia (IHRT) is more effective at improving strength, power, and increasing lean mass than the same training in normoxia.

**Methods:** A pair-matched, placebo-controlled study design included 20 resistance-trained participants assigned to IHRT (FIO2 0.143) or placebo (FIO2 0.20), (*n* = 10 per group). Participants were matched for strength and training. Both groups performed 20 sessions over 7 weeks either with IHRT or placebo. All participants were tested for 1RM, 20-m sprint, body composition, and countermovement jump pre-, mid-, and post-training and compared via magnitude-based inferences.

**Presentation of Results:** Groups were not clearly different for any test at baseline. Training improved both absolute (IHRT: 13.1 ± 3.9%, effect size (ES) 0.60, placebo 9.8 ± 4.7%, ES 0.31) and relative 1RM (IHRT: 13.4 ± 5.1%, ES 0.76, placebo 9.7 ± 5.3%, ES 0.48) at mid. Similarly, at post both groups increased absolute (IHRT: 20.7 ± 7.6%, ES 0.74, placebo 14.1 ± 6.0%, ES 0.58) and relative 1RM (IHRT: 21.6 ± 8.5%, ES 1.08, placebo 13.2 ± 6.4%, ES 0.78). Importantly, the change in IHRT was greater than placebo at mid for both absolute [4.4% greater change, 90% Confidence Interval (CI) 1.0:8.0%, ES 0.21, and relative strength (5.6% greater change, 90% CI 1.0:9.4%, ES 0.31 (relative)]. There was also a greater change for IHRT at post for both absolute (7.0% greater change, 90% CI 1.3:13%, ES 0.33), and relative 1RM (9.2% greater change, 90% CI 1.6:14.9%, ES 0.49). Only IHRT increased countermovement jump peak power at Post (4.9%, ES 0.35), however the difference between IHRT and placebo was unclear (2.7, 90% CI –2.0:7.6%, ES 0.20) with no clear differences in speed or body composition throughout.

**Conclusion:** Heavy resistance training in hypoxia is more effective than placebo for improving absolute and relative strength.

## Introduction

Hypoxia has long been used in combination with endurance training (Terrados et al., 1988). The benefits of endurance training in hypoxia include increased intermittent running performance (Inness et al., 2016), glycolytic enzyme activity (Faiss et al., 2013), and rates of phosphocreatine regeneration (Holliss et al., 2013). Hypoxia may also improve performance in time trials (Czuba et al., 2011), although this is less conclusive (Inness et al., 2016). These benefits are potentially important to enhance team-sport athlete performance.

The development of strength and power is also important for team-sport athletes due to the physical involvements in collision sports including Australian football and the Rugby codes (Dawson et al., 2004; Roberts et al., 2008). Therefore, resistance training plays an integral part in the physical preparation of team-sport athletes. The positive effects of resistance training in hypoxia are becoming evident (Takarada et al., 2000b). When used in combination with resistance training, hypoxia is commonly achieved in two ways, blood flow restriction, and intermittent hypoxic resistance training (IHRT) With blood flow restriction, a pressure cuff is applied to the limb, restricting blood flow, participants then perform resistance training exercises (Takarada et al., 2000b). Blood flow restriction causes many perturbations in the muscle, only one of which is hypoxia. Restricting blood flow to the muscle places the restricted tissue in an ischemic state, leading to hypoxia. Some of the possible mechanisms behind increased strength and hypertrophy through blood flow restriction include increased type ii fiber type recruitment (Yasuda et al., 2010), accumulation of metabolites (Loenneke et al., 2010), increases in plasma growth hormone (Takarada et al., 2000a), and muscle cell swelling (Loenneke et al., 2012).

There are practical limitations to blood flow restriction when using traditional resistance training exercises such as squats, deadlifts, and bench press. Firstly, it is only possible to restrict blood flow to the limbs; therefore a large proportion of musculature used during these exercises is not in a hypoxic state. This causes a disassociation in muscle hypertrophy between the muscles of the limb, which is exposed to blood flow restriction, and the muscles of the trunk, which is not exposed to blood flow restriction (Yasuda et al., 2011). Since blood flow restriction is usually matched with low intensity resistance training, the changes in cross sectional area and strength may not be accompanied by a concomitant increase in connective tissue strength. This is due to decreased mechanical loading through low intensity resistance training used with blood flow restriction (Scott et al., 2015a), therefore the strength of muscles and connective tissue will adapt disproportionately. Increased tensile strength of the tendon might be expected to maintain the safety of the tendon under increasing loads (Buchanan and Marsh, 2002). Although currently unknown, there is a possibility that increasing the strength of the muscle without allowing the tendons time to adapt to this increased force production of the muscle may result in an increased risk of musculotendinous injuries. Due to these limitations in blood flow restriction, it is possible IHRT may be more suited for athletes and strength-trained individuals, as it allows high force production during training that also strengthens connective tissue to aid in injury prevention.

With IHRT, participants perform resistance training in hypoxia, induced by either a normobaric reduction of oxygen content in the mixture (Friedmann et al., 2003; Nishimura et al., 2010), or decreased partial pressure of air as evident at moderate to high altitude (Feriche et al., 2014). There is very little research on IHRT, with conflicting findings on its the effectiveness for increasing 1RM. One of the earliest studies using IHRT showed increased strength and hypertrophy compared to the same training in normoxia (Nishimura et al., 2010). For two sessions per week for 6 weeks, French press and arm curls were performed at 70% 1RM. The IHRT group showed greater hypertrophy and strength than the control group. To further support the use of IHRT, netball athletes undertaking low intensity IHRT had a greater improvement in maximal voluntary contraction than a control group (Manimmanakorn et al., 2013). Similarly, in previously untrained participants, using a moderate intensity resistance training protocol, only the IHRT group improved muscular endurance as measured by maximal repetitions at 70% 1RM (Kon et al., 2014). However, the IHRT group showed no greater improvement in 1RM compared to control (Kon et al., 2014). This is interesting considering a moderate training intensity of 70% 1RM, using 5 sets of 10 reps was used throughout the study.

In the aforementioned study, both groups had similar increases in lean mass and decreases in fat mass (Kon et al., 2014). A low intensity IHRT protocol with previously untrained participants did not have the same effect on muscular endurance (Friedmann et al., 2003). Maximal strength, as measured through a maximal voluntary contraction, did not increase. It is clear the current research is conflicting, and further investigations are required to deduce the best training combinations.

There is currently no research using a high intensity resistance training protocol. The effects of IHRT on strength-trained participants have also not been determined. This study will therefore investigate if heavy IHRT is more effective at developing maximal strength in resistance-trained participants. A secondary aim is to determine whether IHRT aids changes in body composition, sprint performance, and power production during the countermovement jump.

## Methods

### Participants

Twenty strength-trained male participants aged between 18 and 34 volunteered as participants. Participants were required to record a training diary, and qualified as strength trained by achieving at least 12 months continuous resistance training history immediately prior to the study. Resistance training prior to the study needed to include squats and deadlifts as part of their regular training program. The study was approved by the University Human Research Ethics Committee and conformed to the Declaration of Helsinki. All participants provided written informed consent. Participants completed a training log of their current resistance training, including frequency, exercises, sets, repetitions, and intensity to ensure they qualified as strength trained for the purpose of the study. Participants were then pair-matched on absolute 1RM squat strength and training history, and assigned to either the hypoxic (IHRT) or placebo groups.

### Testing

Pre-, mid-, and post-study, participants were scanned using dual energy x-ray absorptiometry (DXA) to assess changes in body composition, tested for 1RM squat strength, countermovement jump, and 20-m sprint time with 5 and 10-m splits.

All participants were familiar with the 1RM squat, a warm up set of five repetitions at 50% predicted 1RM was performed, followed by three repetitions at 80% predicted 1RM, then a single repetition at 90% predicted 1RM. The weight was then increased in small increments until failure, with the goal of achieving a 1RM in a further 3–4 attempts. For the lift to be successful, a depth was required whereby the crease of the hips was below the top of the patella. The same Australian Strength and Conditioning Association Level 3 qualified coach assessed 1RM depth throughout the study. A linear position transducer was connected to the bar, and during the warm-up sets, participants were instructed as to the required depth for 1RM testing by the assessor. This depth was recorded via minimum displacement from the linear position transducer. A failed attempt was recorded if the participant failed to lift the weight, or if adequate depth was not achieved.

The 20-m sprint was performed pre- and post-intervention on the same indoor basketball court using Swift timing gates (Swift, http://www.spe.com.au. Wacol, Queensland, Australia). Participants were instructed to place their toe on a line between the two starting gates, with their weight over their front foot to ensure acceleration occurs from a stationary start with no rock back. Timing gates were placed at 0, 5, 10, and 20-m to record split times. A minimum of three attempts was allowed, with a fourth attempt given if the third trial was the fastest. Participants were offered as much rest as required to ensure each effort was maximal, with a minimum of 3 min given.

A countermovement jump using a force plate, linear position transducer, and corresponding Ballistic Measurement System Software (Fitness Technologies, http://www.fittech.com.au, Adelaide, South Australia, Australia) was used to assess jump qualities. The force plate and linear position transducer were calibrated prior to each testing session according to manufacturer's instructions. Participants performed a familiarization session for the countermovement jump prior to baseline testing. At each testing session, participants performed four single jumps with ~30 s between each jump, with the jump that achieved the highest power output being used for analysis.

Body mass was measured on calibrated scales and height was measured on a calibrated stadiometer. A DXA scan was performed to assess change in body composition measures including fat mass, lean tissue, and bone mass. The DXA scanner used was a Discovery W version 13.4.2. It was calibrated prior to each testing session according to manufacturer's instructions. A standard scanning protocol was used to ensure measurement reliability (Nana et al., 2013). Two experienced technicians performed the scans throughout the study, however each participant was scanned and analyzed by the same technician at pre, mid, and post to remove any inter-tester differences. The protocol for the DXA scan is described in detail elsewhere (Nana et al., 2013). Briefly, participants were positioned on their back in the supine position with hands pronated and legs positioned slightly apart with the femur rotated inwards.

Participants were instructed to maintain a food diary for the first week of the study, and told to replicate this eating plan as closely as possible for the duration of the study. This was to minimize the likelihood that any changes in body composition were due to a change in diet. Participants were instructed not to start taking any supplements.

### Training

During the training sessions, all participants wore a face-mask connected to a hypoxic simulator (Altitude Training Systems, http://www.ats-altitude.com, Lidcombe, NSW, 2141). The hypoxic simulator exposed the participants to simulated altitude by increasing the percentage of nitrogen in the inspired air. The simulator was set at one of two altitudes. The placebo group was exposed to air with an FiO_2_ of 0.20, simulating an altitude of 400-m above sea level, while the hypoxic group was exposed to air with an FiO_2_ of 0.145 for the first 4 weeks, simulating an altitude of 3100-m, and FiO_2_ of 0.141 for the last 3 weeks, simulating 3400-m above sea level. Groups were blinded to their group allocation until the completion of all testing post-study. Participants completed 7 weeks of heavy resistance training three times per week, with sessions performed on non-consecutive days. Each session consisted of squats, deadlifts, and lunges, with repetitions ranging from 3 to 6, and sets ranging from 2 to 4 (Table 1). Rest periods were set at 3 min throughout the study. For squats, starting weight was 75% 1RM for session 1. This weight was chosen after pilot testing, as it was the heaviest weight that could be lifted whereby participants remained blinded to the simulated altitude. During pilot testing, when loads above 75% were used for six repetitions participants were able to correctly guess whether they were in the IHRT or placebo trial. We wanted to ensure participants remained blinded to their groups while still using heavy loads. The placebo effect may play a part in determining endurance changes due to altitude (Lundby et al., 2012), however it is unknown if the placebo effect plays a part in resistance training strength changes through altitude. To control for a possible placebo effect, it was important participants remained blinded to the group allocation. The starting weight for deadlift was the same as squat, while lunge started at 50% squat 1RM. If participants lifted the weight to the predetermined repetition goal, they were encouraged to increase the weight. Participants were asked for a rating of perceived exertion (RPE, Borg 6-20 scale) immediately post-set, and this, combined with the judgment of the researcher was used to determine whether the participant should increase the weight for the next set. This method of increasing the load each session was chosen over a set increase per session or week to allow for individual variations in adaptation over time. For squats and lunges, the bar was positioned on the back across the superior trapezius, with participants instructed to achieve a depth of crease of hips below the top of the patella, as for 1RM testing. For deadlift, the weight was lowered to the ground between each rep. As participants were experienced in these exercises, a degree of flexibility was allowed regarding placement of feet, with some choosing a wider stance, and others a narrower stance.

**Table 1:** **Repetitions (Reps) and sets each week for squats, deadlifts, and lunges (3 lunges each leg per set) and percentage 1RM for squat lifted and post-set RPE for each group each week**.

**Week**	**Sets**	**Squat reps**	**Deadlift reps**	**Lunge reps**	**Squat %1RM IHRT load**	**Squat %1RM placebo load**	**Post-set IHRT RPE**	**Post-set placebo RPE**
1	3	6	6	6	77.5±3.7	78.2±3.9	6.74±1.63	6.02±2.19
2	3	5	5	6	85.4±5.3	83.8±4.0	7.39±2.14	7.03±1.90
3	3	4	4	6	94.4±5.6^#^	90.5±4.1	7.80±2.28	7.38±2.13
4	2	4	4	6	99.2±5.4^#^	94.2±4.5	6.98±2.44	6.71±2.16
5	3	5	5	6	100.4±6.5^#^	94.7±5.9	8.14±2.49	7.66±2.10
6	4	4	4	6	104.2±7.8^#^	98.6±6.5	7.86±2.80	7.71±1.87
7	4	3	3	6	109.9±8.2^#^	104.5±5.9	7.60±2.80	7.83±2.15

# Likely Large effect in the difference of 1RM lifted between groups. All data is Mean ± SD.

The hypoxic mask was worn for the last warm up set and all working sets. Once applied, the hypoxic mask was not removed until the completion of the session (~45 min).

Prior to hypoxic exposure, baseline SpO_2_ and heart rate were taken via pulse-oximetry. These values were also taken pre- and post-each working set, with the values immediately prior to the set used as the pre-set value, and the lowest value of SpO_2_ and highest value for heart rate used as the post-set value. Post-set values were usually achieved 15–30 s post-set. As well as the 6–20 Borg scale post each working set, the Borg 1–10 RPE scale was used post-session. After the first session, and regularly throughout the 7 weeks, participants were asked which group they thought they were in.

### Statistical analysis

A contemporary statistical approach was used to analyse all data, and expressed as mean ± *SD* and effect size [ES ± 90% confidence limits (CL)]. Percentage change was determined in comparison to baseline. The difference in the change between groups was determined using both ES and % changes ± 90% CL. Where the difference in between group change for 90% CL crossed from positive to negative (across 0%), this was interpreted as unclear at 90% CL. Standards for measuring ES were as previously described (Hopkins et al., 2009).

## Results

Participants were blinded to condition, with only one participant guessing their group allocation. Table 2 gives details of the participants. Of the 20 volunteers, 18 completed the study (9 per group), while the other two completed the mid testing. One participant withdrew due to illness, and the other through injury, both unrelated to the training study. Only the 18 who finished the study were included for post-analysis, while all 20 participants were included in the mid testing analysis. Groups were not clearly different for any of the testing procedures at baseline.

**Table 2:** **Testing results for all performance tests for both IHRT and Placebo groups**.

**Test**	**IHRT group**			**Placebo group**		
	**Pre (*n* = 10)**	**Mid (*n* = 10)**	**Post (*n* = 9)**	**Pre (*n* = 10)**	**Mid (*n* = 10)**	**Post (*n* = 9)**
Height (cm)	183.1±4.5	183.1±4.5	184.1±4.5	181.0±5.8	181.0±5.8	180.8±5.8
Body mass (kg)	83.1±7.5	83.0±7.5	83.7±7.9	80.2±12.0	80.5±11.2	78.7±9.8
Absolute 1RM strength (kg)	121.4±22.1	138.2±27.8	148.4±32.7^**^	125.5±30.7	135.7±29.5	141.8±28.8^#^
Relative squat strength (kg.bm^−1^)	1.46±0.19	1.66±0.22	1.76±0.26^**^	1.56±0.30	1.69±0.29	1.80±0.25^#^
5 m split time (s)	1.17±0.08		1.17±0.03	1.13±0.08		1.12±0.07
10 m split time (s)	1.94±0.11		1.94±0.06	1.89±0.09		1.88±0.08
20 m split time (s)	3.25±0.15		3.27±0.10	3.21±0.14		3.18±0.09
Absolute Peak Power (W)	4360±602		4676±463^*^	4333±656		4299±527

* Possibly greater pre-post change than placebo.

** Likely greater change than placebo.

# Likely greater than pre. All data is Mean ± SD.

### Baseline

Absolute strength was 121.4 ± 22.1 kg for IHRT and 125.5 ± 30.7 kg for placebo. Relative strength was 1.46 ± 0.19 kg.bm^−1^ for IHRT and 1.56 ± 0.30 kg.bm^−1^ for placebo. Differences between groups for strength, or any other testing parameter at baseline were unclear (Table 2).

### Training data

Pre-training SpO_2_values were not different between groups (98.3 ± 1.3% for IHRT and 98.4 ± 1.3% for placebo; Table 3). During the session, oxygen desaturation occurred in IHRT only, with a *likely* large effect in the difference between groups pre-set (90.0 ± 2.5% for IHRT vs. 97.3 ± 1.3% for placebo. ES −5.61, 90% CL for ES −5.7 to −5.5). There was also greater desaturation post-set for IHRT with a *most likely* large effect in the difference between groups (84.1 ± 3.5% for IHRT vs. 96.5 ± 1.7% for placebo, ES −7.3, CL for ES −7.4 to −7.2; Table 3). Neither session RPE (IHRT 7.5 ± 1.3 vs. placebo 7.3 ± 1.6), nor post-set RPE (IHRT 15.4 ± 2.5 vs. placebo 15.2 ± 2.3) were different between groups.

**Table 3:** **Average SpO_2_ and Heart Rate values pre and post all sets for the duration of the training study and post-set RPE for each group**.

	**IHRT pre-session**	**IHRT pre-set**	**IHRT post-set**	**Placebo pre-session**	**Placebo pre-set**	**Placebo post-set**
SpO_2_%	98.29±1.3	89.97±2.5^*^	84.13±3.5^*^^#^	98.38±1.3	97.26±1.3	96.51±1.7
Heart Rate (BPM)	93.8±19.1	103.7±19.6	144.8±19.0^**^	93.8±17.2	104.0±18.3	144.9±18.2^**^
RPE (au)			15.43±2.5			15.16±2.3
Post-session RPE (au)			7.53±1.28			7.27±1.61

* Most likely less than placebo.

# Most likely less than pre.

** Most likely greater than pre-set. All Data is Mean ± SD.

Heart rate was higher post-set compared to pre in both groups (pre- to post-set HR 103.7 ± 19.6 to 144.8 ± 19.0 bpm for IHRT, and 104.0 ± 18.3 to 144.9 ± 18.2 bpm for placebo), with no clear difference between groups (Table 3). Load lifted when reported as percentage of squat 1RM was not different between groups during week 1 (77.5. ± 3.7% for IHRT vs. 78.2 ± 3.9 for placebo). For squat, there was a trend toward IHRT lifting a greater percentage of baseline 1RM compared to placebo throughout the study (Table 3). By week 3, IHRT was lifting a greater percentage of starting 1RM for squat compared to placebo, with a *likely* large effect (3.9%, ES 0.94, 90% CL 0.65–1.24). This greater percentage of 1RM lifted in training for IHRT compared to placebo remained through to week 7 (5.4%, ES 0.91, 90% CL 0.61–1.20).

### Mid

**Figure 1:**
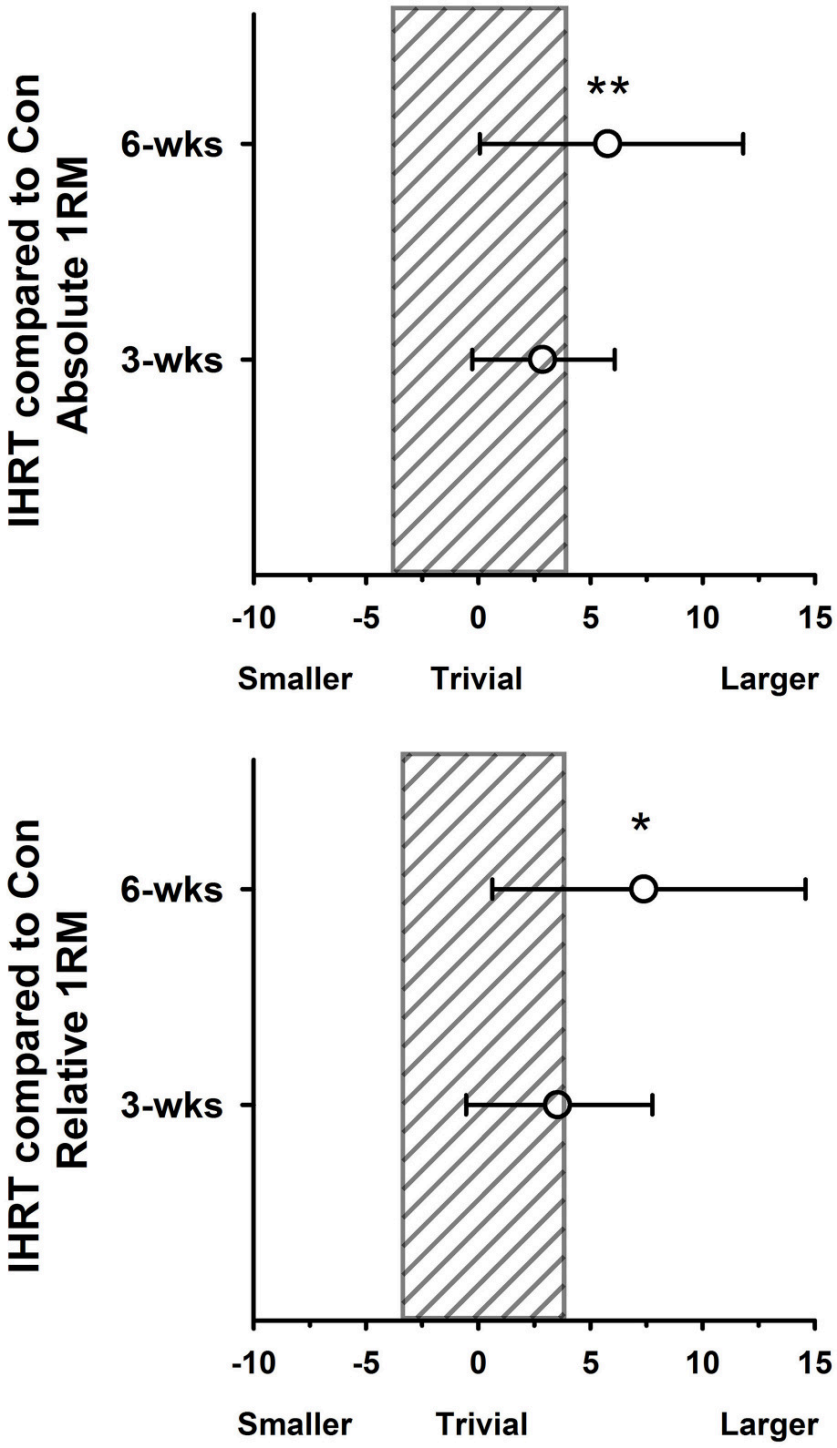
**Percentage difference in the change in performance from baseline for intermittent hypoxic resistance training (IHRT) compared with placebo with 90% CI**. **(A)** Between-groups change in Absolute 1RM strength. **(B)** Between-groups change in Relative 1RM strength. Shaded bar represents uncertainty in the measure. ^*^Possibly small effect in the difference in change between groups, ^**^Likely small effect in the difference in the change between groups.

Compared to baseline, both groups improved both absolute (12.7 ± 3.9% for IHRT vs. 8.7 ± 4.7% for placebo) and relative (12.9 ± 5.1% for IHRT vs. 8.2 ± 5.3% for placebo) 1RM squat (Figure 1). Compared to placebo, the IHRT group had a *possibly* greater change for absolute (4.4% greater change, CL 1.0–8.0%, ES 0.21, 90% CL 0.05–0.37) and a *likely* greater change for relative 1RM (5.1% greater change, CL 1.0–9.4%, ES 0.31, 90% CL 0.06–0.57). There was no change for lean mass or countermovement jump parameters either between or within groups.

### Post

Compared to baseline both groups improved both absolute (22.2 ± 7.6% for IHRT vs. 14.2 ± 6.0% for placebo) and relative (20.5 ± 8.5% for IHRT vs. 13.6 ± 6.4% for placebo). The IHRT group had a *likely* greater change than placebo from pre to post for both absolute (7.0% greater change, CL 1.3–13.0%, ES 0.33, 90% CL 0.06–0.59) and relative (8.0% greater change, 90% CL 1.6–14.9%, ES 0.49, 90% CL 0.10–0.87) 1RM. At post, only IHRT improved countermovement jump peak power, however this change was unclear in comparison to placebo (2.7% greater change, 90% CL −2.0–7.6%, ES 0.20, 90% CL −0.15–0.54). There was no difference between or within groups compared to pre for 20-m sprint or body composition.

## Discussion

Hypoxia improved both absolute and relative 1RM compared to the same training using a placebo despite no change in lean mass. The placebo group also improved 1RM strength, however the IHRT change was 5.8 and 9.2% greater than placebo in absolute and relative 1RM strength, respectively. Considering participants were already strength trained with a moderate to high level of strength, this is a meaningful change between groups after only 7 weeks.

This is the first study to use heavy resistance training combined with hypoxia. Only an acute resistance exercise bout has been previously used (Scott et al., 2015b), therefore the present study reports original findings using heavy resistance training and hypoxia in a training study. The majority of resistance and systemic hypoxia studies used low to moderate intensity resistance training of ~30–70% 1RM. However, the present study used 75% 1RM for the first session, increasing to 104% starting 1RM for placebo and 109% for IHRT. Unlike high-intensity interval training in hypoxia, which shows a decrease in maximal work (Balsom et al., 1994), hypoxia does not appear to impact on physical performance during high-load resistance exercise. This is shown by no differences in RPE and heart rate between groups, and is in agreement with the only other research using heavy resistance exercise and hypoxia. Using five repetitions of squat and deadlift in three different conditions (FiO_2_ of 0.21, 0.16, and 0.13), heart rate, and post-set RPE were not different in the conditions with an FiO_2_ of 0.21 and 0.16 despite using the same load. However, at the more extreme hypoxia, heart rate was higher than the other two conditions (Scott et al., 2015b). This, combined with our findings, confirms resistance training exercise intensity is not affected by moderate hypoxia, at least in trained individuals.

While increased strength and hypertrophy is consistently achieved through resistance training combined with blood flow restriction (Takarada et al., 2000b; Scott et al., 2014), there are conflicting findings whether systemic hypoxia increases strength to a greater extent than resistance training alone. The inconsistent findings can be partly attributed to differences in study design. For example, studies of moderate volume and intensity (3–4 sets of ~70% 1RM) generally show greater changes in muscle hypertrophy using hypoxia (Nishimura et al., 2010; Kurobe et al., 2015), although one showed no difference between groups (Kon et al., 2014). There are also inconsistent results regarding changes in 1RM between groups when using IHRT with submaximal loads. Studies show increases in 1RM using both moderate (Nishimura et al., 2010) and very light loads (Manimmanakorn et al., 2013), while others show no benefit on 1RM through hypoxia compared to control (Kon et al., 2014). Unfortunately, the study by Nishimura et al. used a predictive equation to determine 1RM from a 10RM test. Therefore, although authors concluded an increase in muscular strength, it is rather likely that, because of the non-specific testing (Reynolds et al., 2006; Tanner and Gore, 2013), an increase in muscular endurance explained the change in predicted 1RM.

Two studies have used tests of moderate intensities to test for muscular endurance, similar to the load used during training, with hypoxic groups showing both a benefit (Kon et al., 2014), and no change in performance (Kurobe et al., 2015). In the Kon et al. study, systemic hypoxia showed no greater change in 1RM compared to control, however when testing using the same intensity as training, muscular endurance improved more in the hypoxic group (Kon et al., 2014), showing the importance of the testing protocol matching the training protocol.

As all hypoxia and resistance training studies to date have used moderate intensities more suited to hypertrophy and muscular endurance gains (Fleck and Kraemer, 2014), combined with the differences in methodology for determining 1RM, it is not surprising that changes in 1RM through systemic hypoxia are conflicting. Therefore, it is important to ensure the testing battery is appropriate to reflect the nature of the training intervention (Tanner and Gore, 2013). Systemic hypoxia may merely magnify the expected outcome from a given training program, with specificity of the stimulus and testing protocol important to test the outcome of training.

It is possible there were no increases in lean mass due to our study employing a lower volume, high intensity program designed to increase maximal strength more so than muscle hypertrophy. This type of protocol is more likely to see changes in strength as opposed to muscle hypertrophy (Kraemer and Ratamess, 2004). This change in strength despite a lack of change in lean mass is an important finding, as many athletes, including team-sport athletes with a high running demand in their sport, athletes competing in weight classes, and many endurance athletes want to increase strength without an increase in mass.

Other researchers have concluded that the placebo effect may be at least partly responsible for performance changes through hypoxia (Siebenmann et al., 2012), however this was not the case in our study. As participants were successfully blinded to the environmental condition, the greater changes in strength observed in IHRT in the present study cannot be attributed to a placebo effect. Due to no placebo effect, and no changes in lean mass, we cannot fully explain the mechanisms responsible for the differences between groups. When beginning strength training, most of the early strength changes can be attributed to neural adaptations (Moritani and deVries, 1979). Such early changes include adaptations in agonist, antagonist, and stabilizer muscle activation, increased firing frequency, motor unit synchronization, and agonist co-activation (Folland and Williams, 2007). As all participants were strength trained, matched for training status and 1RM, any neural adaptations could be expected to be minimal.

When undertaking endurance training in hypoxia, intermittent hypoxic training maintains the proportion of type II fibers to a greater extent to the same training in normoxia (Zoll et al., 2006). After 6 weeks, an intermittent hypoxic training group had the same percentage of type II fibers (29.4 ± 7.4 at pre, and 29.9 ± 6.2 at post), whereas the same training in normoxia saw type II fibers decrease (41.0 ± 8.1% at pre, to 33.9 ± 6.6% at post; Zoll et al., 2006). Type II muscle fibers have a greater force production than type I fibers (Bottinelli et al., 1999). As intermittent hypoxic training maintains type II fibers, if this maintenance of type II fibers is also apparent with IHRT, it is possible there was a greater strength adaptation in the IHRT group compared to placebo despite no changes in muscle mass due to type II fiber maintenance.

The decreases in SpO_2_ in IHRT are similar to other systemic hypoxic studies (Kon et al., 2010; Scott et al., 2015b). A decrease in SpO_2_ occurs with hypoxia, and this activates a cascade of events that eventually lead to changes in endurance performance (Rusko et al., 2004). It is yet to be determined what effect a decrease in SpO_2_ has on changes seen through IHRT. There is reduced central fatigue following adaptation to hypoxia (Amann et al., 2013). Enhanced cerebral O_2_ delivery to compensate for hypoxia could enhance neurotransmitter turnover, thus enhancing skeletal muscle fiber firing rate (Amann et al., 2013), which is a typical neural adaptation seen through resistance training. The heavy resistance training used in the current study increases the level of neural activity (Tan, 1999). Whether these changes are evident following IHRT is unknown, however if apparent, this would possibly explain an adaptation to hypoxia that may increase strength through neural changes including muscle fiber firing rate. Although there are possible neural changes that could explain increases in 1RM through IHRT, most changes in neural adaptation occur quite early in a resistance-training program (Moritani and deVries, 1979). As we used resistance-trained participants, most of the neural changes would have already occurred. Therefore, it is quite surprising that there were changes in 1RM despite no change in muscle mass. Because of this, the mechanisms behind increased 1RM are not known. Neural changes should be analyzed in further IHRT studies to determine whether neural changes are responsible for the change in performance following IHRT.

We found no change in 20-m sprint performance for either group, despite a change in 1RM. Although only IHRT improved CMJ peak power, the difference in the change between groups was unclear. There is a strong correlation between maximal squat strength and sprint performance (Wisløff et al., 2004; McBride et al., 2009), this correlation is also apparent between maximal squat strength and CMJ performance (Wisløff et al., 2004). It is also generally believed that improving 1RM directly increases sprint performance. In soccer athletes, there was a small change in 20-m sprint times after improvements in 1RM squat strength (Styles et al., 2016). A low volume, heavy resistance training protocol was used. Although not stated, the Styles et al. study was performed during the competition season and it is assumed these athletes would have been exposed to maximal running velocity during training and matches. Therefore, strength changes through resistance training, combined with the sprint training during the on pitch training may have combined to increase 20-m sprint performance. To support this, strength training only, and sprint training only displayed the same changes in 30-m sprint times, while a combination of strength and sprint training had a greater enhancement in 30-m sprint times (Marques et al., 2015).

As our study was heavy resistance training only, and the participants were not performing sprint training as part of their normal activity outside of the study, a possible reason for the lack of change in 20-m sprint times and CMJ performance in our study is due to no explosive training being performed as part of training. In a study on team-sport athletes, a group that performed plyometric exercises improved sprint times, while a control group showed no change (Marques et al., 2013), however it should be noted that neither group performed resistance training as part of the study. The addition of sprint or plyometric training combined with maximal strength training may therefore be important to improve sprint times, with resistance training alone insufficient to increase speed and power.

In a normal periodized resistance-training program for athletes, high velocity resistance training either follows, or is completed in conjunction with heavy strength training. This change in strength could well be transferred into changes in power with appropriate subsequent training.

## Practical applications

- IHRT increases relative and absolute 1RM in comparison to a strength and training matched control group.
- Increases in 1RM occurred despite no changes in muscle mass, 20-m sprint or CMJ parameters.
- Athletes wanting to increase strength without increasing muscle mass are advised to undertake heavy resistance training in systemic hypoxia.

## Author contributions

MI, FB, RA responsible for study design, recruitment of participants, data collection, data analysis, manuscript drafting and editing. EW, AP, AS responsible for some data collection, some data analysis and editing manuscript.

### Conflict of interest statement

The authors declare that the research was conducted in the absence of any commercial or financial relationships that could be construed as a potential conflict of interest.
